# Clinical Significance of Disseminated Pluripotent Tumor Cell Signature Expression in the Bone Marrow from Patients with Colorectal Cancer

**DOI:** 10.4172/1948-5956.1000490

**Published:** 2017

**Authors:** Martin Gasser, Mia Kim, Roberta Rehder, Natasha Frank, Markus Frank, Tanja Grimmig, Romana Moench, Carmen Ribas, Bertram Illert, Christoph-Thomas Germer, Andreas Rosenwald, Ana Maria Waaga-Gasser

**Affiliations:** 1 Department of Surgery I, University of Wuerzburg, Wuerzburg, Germany; 2 Medical School, Evangelic Faculty of Paraná, Curitiba, PR, Brazil; 3 Transplantation Research Center, Children’s Hospital Boston, Harvard Medical School, Boston, USA; 4 Department of Surgery I, Molecular Oncology and Immunology, University of Wuerzburg, Wuerzburg, Germany; 5 Institute of Pathology, University of Wuerzburg, Germany; 6 Transplantation Research Center, Brigham and Women’s Hospital, Harvard Medical School, Boston, USA

**Keywords:** Disseminated tumor cells, Cancer initiating cells, Bone marrow, *ABCB5*, Colorectal cancer

## Abstract

**Purpose:**

Disseminated tumor cells (DTCs) are critically involved in tumor relapse and survival in several invasive tumors. We previously showed that the ATP-binding cassette (ABC) transporter, *ABCB5*, is a chemoresistance mediator expressed on specific cell subsets in colorectal cancer (CRC) and other malignancies. This study evaluated the molecular signature expression and its clinical relevance of DTCs in bone marrow from patients with colon cancer.

**Methods:**

This study included 49 consecutive patients (UICC stage I-IV) that underwent curatively intended or palliative surgery for CRC. We analyzed cells from bone marrow aspirates obtained before surgery and derived from patients that had completed minimally a 5-year follow-up. The gene expression of *ABCB5* in comparison to *CD133* (molecule for identifying cancer initiating cells), *Lgr5* (an intestinal stem cell marker) as well as Cytokeratin (CK) 20 (terminally differentiated tumor cells of epithelial origin) in these cells was evaluated.

**Results:**

Bone marrow analysis showed differential expression between the analyzed genes. *ABCB5* and *Lgr5* and to lesser extent *CD133* and *CK20* genes were significantly expressed in the analyzed cells from bone marrow aspirates while only *ABCB5* and *Lgr5* were significantly negative associated with tumor progress and overall survival.

**Conclusion:**

Overexpression of *ABCB5* and *Lgr5* in bone marrow negatively influenced patient survival pointing to a specific chemo resistant and pluripotent cell subgroup of DTCs in the bone marrow. *ABCB5* like *Lgr5* positive cells seem to be involved in limited tumor related patient survival, suggesting that *ABCB5-* and *Lgr5*-positive cells may be relevant for specific clinical intervention strategies

## Introduction

Colorectal cancer (CRC) is the second most commonly diagnosed type of cancer and the second most common cause of cancer-related death in developed countries [1]. The 5-year survival rate of patients diagnosed with advanced stage IV disease is less than 25% [2] and almost half of the patients, who undergo curative resection, ultimately die of metastatic or recurrent disease as a result of residual microscopic disease not evident at the time of surgery [3,4]. This fact strongly suggests that the dissemination of metastatic cells from the primary solid tumor to distant sites can occur early during disease progression [5–7]. A minority of tumor cells with different thus pluripotent characteristics, so called cancer initiating cells (CICs) also referred to as cancer stem cells, is capable of initiating and maintaining tumor growth. Resistance of these CICs against radio- or chemotherapy could explain tumor relapse after years. Studies have shown that colon CICs are more resistant to treatment with 5-FU or oxaliplatin [8,9] and when colon cancer cell lines were treated with 5-FU or oxaliplatin *in vitro*, an increase of the CIC fraction could be observed [10]. The discovery of reliable markers that identify CICs will pave the way to a better understanding of regulatory mechanisms that determine stemness, CIC differentiation, and therapeutic target mechanisms. Some of the most frequently applied markers used to detect DTCs in the bone marrow of cancer patients include the cytokeratins (CKs). CKs are a group of markers expressed at various levels in epithelial cells [11,12] that under normal circumstances should not circulate unless they become metastatic. The *CK20* gene is mainly expressed in gastric and intestinal epithelium, urothelium and Merkel cells [13]. Its expression has also been observed in colorectal cancer [14–16]. Until now several different markers for the enrichment of CICs are described. The five-transmembrane glycoprotein *CD133* is one of the first colon CIC markers identified. After application of Oxaliplatin, 5-FU or Cyclophosphamide this cell fraction was enriched *in vitro* and *in vivo* pointing to a specific role in chemo resistance [17,18]. Not only cell surface markers, but also activity of certain pathways or enzymes can mark stemness. The Wnt signaling activity can serve as a functional designation of colon CICs [19]. It is interesting to note that Leucin-rich-repeat-containing G protein-coupled receptor 5 *(Lgr5)* as a Wnt target gene is expressed on normal intestinal stem cells and in human colon CICs [20,21]. Another potential CIC target is *ABCB5*, a novel human multidrug resistance mediator recently shown to be expressed by cells of melanocytic lineage and additional cancers responsible for resistance to chemotherapy *in vitro* [22,23]. Subsequent work has shown that *ABCB5* identifies CICs in malignant melanoma that correlate with clinical disease progression and that can be specifically targeted to abrogate tumor growth [22]. Recently, we have shown that *ABCB5* is expressed at higher levels in clinical CRC compared to healthy colonic mucosa, and that the *ABCB5*-positive tumor cell population was significantly induced following 5-Fluorouracil therapy in CRC patients’ tissue [24], demonstrating their relevance as therapy resistant cell fraction. In this study we analyzed *ABCB5, CD133, Lgr5*, and *CK20* expression in bone marrow cells from patients with CRC and with completed 5-year follow up. This could lead to more directed therapies which specifically target therapy resistant tumor cells in CRC patients.

## Materials and Methods

### Patients

Consecutive patients undergoing elective or palliative surgery for primary colorectal cancer (n=670) at the University Hospital of Wuerzburg were prospectively enrolled from July 2003 until June 2008 in this study. Of these, 49 patients with consent for bone marrow sample aspiration were included in this study. Patients with secondary carcinoma of other origin were excluded. With a median follow-up period of 58 months (range 4–141), all patients were followed-up regularly at 3 to 6-month intervals in accordance with the guidelines of the German tumor centers (completeness index of 0.96) [4]. Tumors were evaluated for location, stage, and differentiation grade. Data concerning age, gender, level of wall infiltration, and lymph node metastasis were collected in the database of the Wuerzburg Comprehensive Cancer Center Registry. The study was approved by the regional ethical committee.

### Bone marrow analysis

After the induction of general anesthesia prior to primary tumor surgery, bone marrow samples (7–12 mL) were obtained by aspiration from both iliac crests. After density centrifugation through Ficoll-Paque (Pharmacia, Freiburg, Germany; 30 min, 400 × g) mononuclear cells were harvested and washed twice in phosphate-buffered saline. Cell pellets were snap-frozen in liquid nitrogen and stored at −80°C until further use.

### RNA extraction and cDNA synthesis

RNA extraction from bone marrow samples were lysed in Trizol reagent (Invitrogen, Darmstadt, Germany) according to the manufacturer’s recommendations. cDNA synthesis was carried out by using iScript cDNA Synthesis Kit (Bio-Rad, Hercules, CA, for bone marrow cells and Promega, Mannheim, Germany, for peripheral mononuclear cells) as described by our group [25].

### Real time quantitative reverse transcription PCR (RT-qPCR)

*ABCB5*, *Lgr5*, *CD133*, and *CK20* gene expression was analyzed in bone marrow samples (n=49) from colon cancer patients and healthy control individuals (n=20) by RT-qPCR assay. Total RNA (1 μg) was incubated with DNase I and reverse transcribed with oligo(dT) with SuperScript II RT-PCR (Promega). Reverse transcriptase product (100 ng) was amplified by primer pairs specific for *Lgr5* (QT00027720), *CD133* (QT00075586) and *ABCB5* (5V-CACAAAAGGCCATTCAGGCT-3V, forward) and 5V-GCTGAGGAATCCACCCAATCT-3V, reverse) which were purchased from Qiagen (Hilden, Germany). Beta-actin was used as a normalizing control. Relative gene expression was measured with the GeneAmp 7000 Sequence Detection System (Applied Biosystems). The average value of both duplicates was used for calculation of relative transcript quantity according to the ΔCt method, as described previously [26]. Results from bone marrow samples were compared to those from healthy individuals by calculating the difference between the average ΔCt values (ΔΔCt) and the relative fold difference as 2^−ΔΔCt^. Upregulated gene expression, described as positive expression in Tables 1 and 2, of *ABCB5, Lgr5, CD133*, and *CK20* in bone marrow was defined on the definition of ≥ 1.5 fold difference compared to the gene expression determined in controls.

### Statistical analysis

The data collected were analyzed using SPSS for Windows version 12.0 (SPSS, Munich, Germany). Differences between groups were analyzed using Chi square or Fisher’s Exact test. The calculation of survival rates and overall survival were performed using the Kaplan-Meier method. Disease free survival was calculated from the date of surgery to the date of tumor relapse, and overall survival from the date of surgery to the date of the patient’s death. Patients without evidence of disease recurrence were censored at the date of last contact, as a small number of patients who were lost to follow-up. Factors such as age, UlCC-stage (pTNM), and gene expression for *ABCB5*, *Lgr5*, *CD133*, and *CK20* determined by quantitative Real Time PCR analysis were examined using univariate analysis. Statistical differences in univariate analysis were calculated using the Log-rank test. A p value<0.05 was considered statistically significant._

## Results

### Characteristics of the study population

**Figure 1:**
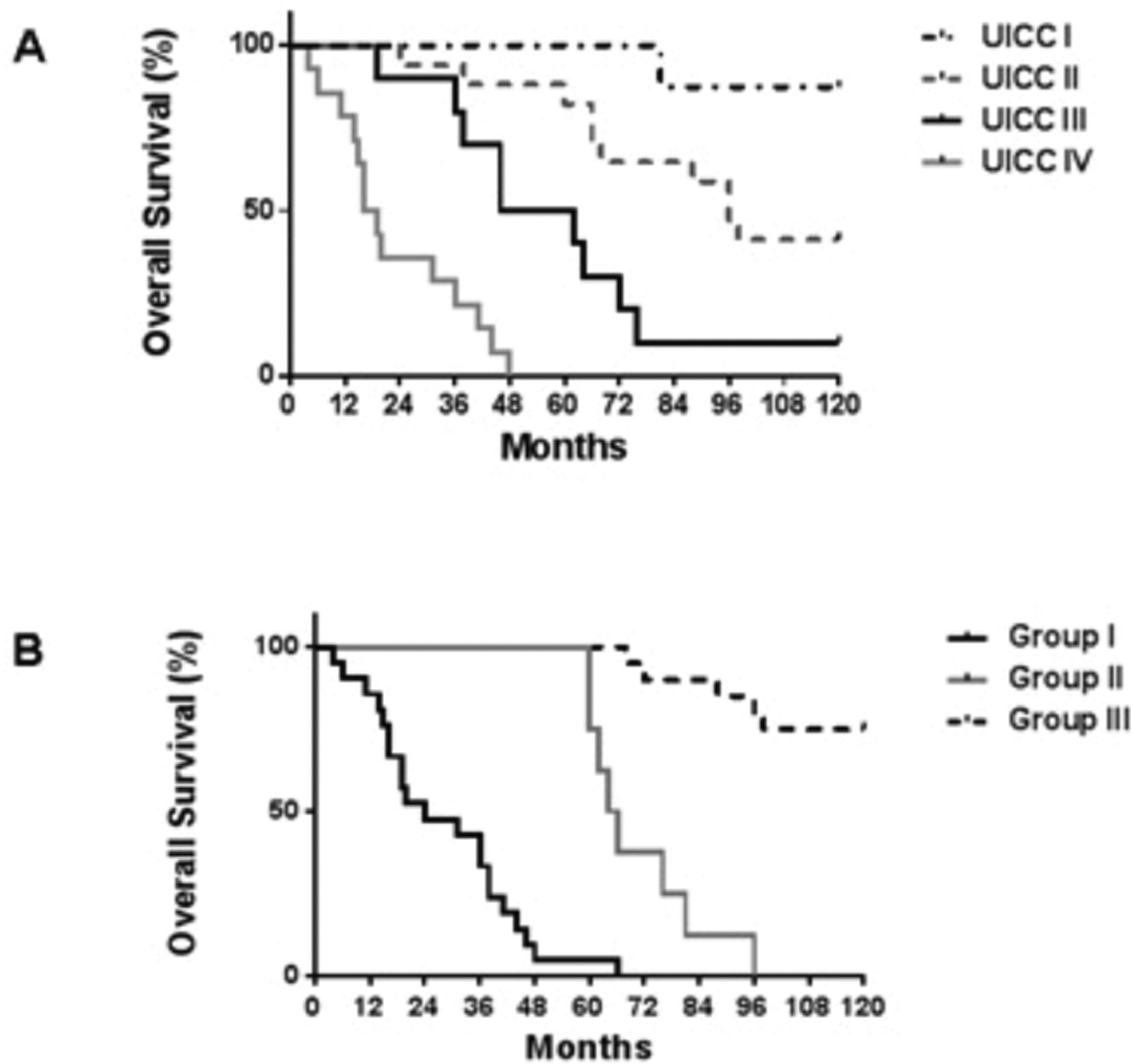
Kaplan Meier analysis of the study population with regard to UICC stage (A) and tumor relapse and survival (B). Group I patients developed either a tumor relapse or were characterized by early tumor progress and finally died related to their tumor disease; Group II patients developed a tumor relapse but survived long-term (≥ 60 months) after a second surgery for their metastasis without another tumor relapse; and Group III patients survived long-term (≥ 60 months) and never developed a tumor relapse.

The patient and tumor characteristics are presented in Table 3 and Figure 1A. The analysis included 19 male patients (38.8%) and 30 female patients (61.2%), and the median age was 68.7 years (range 41–82). All patients were diagnosed with colon cancer (adenocarcinoma). Histologically, two tumors (4.1%) were well differentiated, 31 (63.3%) were moderately differentiated, 16 (32.7%) were poorly differentiated, and no tumors were mucinous. Using the 7^th^ UICC TNM staging system, 3, 3, 33 and 10 patients had stage I, II, III and IV cancers, respectively. Among the analyzed patients 82% and 90% of the individuals significantly expressed *ABCB5* and *Lgr5*, while 71% and 59% showed upregulated expression for *CD133* and *CK20*, suggestive for the existence of DTCs in this compartment (Tables 1 and 2).

### Correlation of clinical and pathologic features with CICs

**Figure 2:**
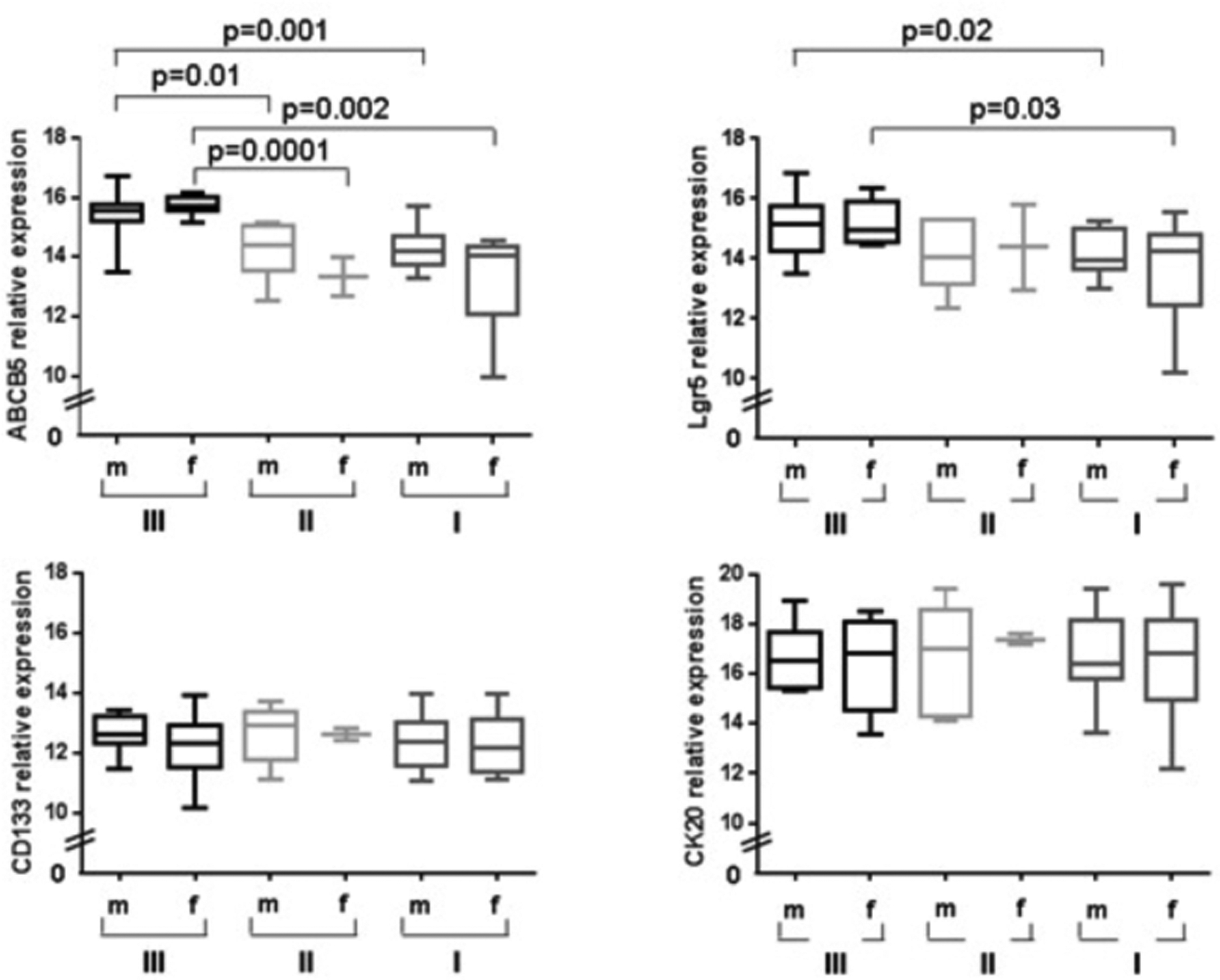
*ABCB5, Lgr5, CD133*, and *CK20* gene expression analysis in bone marrow of patients with colon cancer, differentiated between patients that developed either a tumor relapse or a tumor progress and finally died related to their tumor disease (m for males and f for female patients, Group I); patients that developed a tumor relapse but survived long-term (≥ 60 months) after a second surgery for their metastasis without another tumor relapse (Group II); and patients that were long-term survivors and never developed a tumor relapse (Group III).

Each transcript was tested in its dependency from gender, age, differentiation, T-, N-, and M-category, and UICC-stage. Transcript-positive patients were compared to transcript-negative patients. Expression of *ABCB5* and *Lgr5* in the bone marrow was dependent on tumor grading (both p=0.01) and T-category (p=0.036 and p=0.031, respectively). Significantly increased expression was shown in most of the samples for *ABCB5* and *Lgr5* and to lesser extent for *CD133* and *CK20* (listed as positive expression in Table 1). Clinicopathological characteristics demonstrated a stage-dependent process of upregulated genes (Table 1). Differences in gene expression profiles of *ABCB5, Lgr5, CD133*, and *CK20* dependent on the underlying tumor disease and thus on each T, and UICC stage was additionally compared between three different groups of patients. Group I developed either a tumor relapse or were characterized by early tumor progress and finally died related to their tumor disease (Figure 1B); Group II developed a tumor relapse but survived long-term (≥ 60 months) after a second surgery for their metastasis without another tumor relapse; and Group III were long-term survivors (≥ 60 months) and never developed a tumor relapse. *ABCB5* expression in the bone marrow from the group of patients that developed either a tumor relapse or a tumor progress and finally died related to their tumor progress (Group I) was significantly upregulated when compared to patients from Group III that represented long-term survivors without any tumor relapse (Figure 1 upper left graph, group I versus III each for males and females, p=0.001 and 0.002, respectively (Figure 1B). Patients from Group II demonstrated comparable results (group II versus group III each for males and females, p=0.01 and 0.0001, respectively). Interestingly, comparable results were shown for *Lgr5* (Figure 2 upper right graph, group I versus group III, p=0.02 and 0.03) but not for *CD133* or *CK20* expression (lower left graph for *CD133*, and lower right graph for *CK20*). In addition, most patients with a cluster of genes for *ABCB5, Lgr5*, and *CD133* were derived from group I that developed metastases and/or died tumor related. However, only co-expression of the two genes *ABCB5* and *Lgr5* but not of *CD133* correlated with patient prognosis (Group I: *ABCB5* plus *Lgr5* subgroup=23.2 months vs. *ABCB5* plus *Lgr5* plus *CD133* subgroup=26 months, p=0.01; mean: 28 months for all group I patients).

### Overall survival and relapse-probability

*ABCB5*, *Lgr5*, *CD133*, and *CK20* gene expression in bone marrow collected from patients with colon cancer was further quantified and tested for their influence on overall survival in the patients. Patients overexpressing *ABCB5* and *Lgr5* but not *CD133* nor *CK20* in their bone marrow showed a worse overall survival compared to biomarker-negative patients (p=0.022, and p=0.049 for *ABCB5* and *Lgr5* versus p=0.529, and p=0.683 for *CD133* and *CK20*, respectively) (Table 1).

## Discussion

The prognosis of patients with colorectal cancer is largely determined by the occurrence of distant metastases and the fact of chemo resistance during tumor progression. Relapse is mainly due to clinically occult micro metastasis present but not detectable in secondary organs as well as compartments like peripheral blood and bone marrow at the time of primary diagnosis. Bone marrow of patients after R0-resection of solid tumors are currently discussed to be a common homing site of remaining disseminated and circulating tumor cells. Beside the ongoing discussion of the source of tumor cells responsible for tumor relapse after primary cure, recent evidence suggests a specific side population of cells with a distinct self-renewal capacity and chemo resistance, which is gaining increasing interest in the field of DTCs. Thus, it seems to be possible that such a subset of chemo resistant cells with self-renewal capacity within a ‘specific niche’ as the bone marrow may be a major source for tumor maintenance. Moreover, this tumor cell dissemination can be an early event during tumor progression. Recent evidence has suggested that a specific subpopulation of chemo resistant cells may be a major source of tumor maintenance. Nevertheless, there is no clear evidence so far for such a cell subset derived from the primary tumor. This may be of highly relevance for adjuvant therapies when detected even before its complete resection. Our findings suggest that a subset of therapy-refractory cancer cells may have prognostic significance. Gene signature expression of these cells was identified in the bone marrow of patients with CRC. This indicates such a subset of DTCs with putative self-renewal and chemo resistant characteristics among DTCs and its prognostic significance. mRNA expression for *ABCB5* and *Lgr5* when detected in the bone marrow significantly influenced overall survival. Thus, it implicates a subset of specific tumor cells migrating from the primary tumor during tumor progression into the bone marrow and becoming part of future tumor recurrence. That means that dissemination of therapy-refractory cancer cells into a ‘niche’ within the bone marrow would enable the tumor to progress at later time after primary surgery when such cells re-start to proliferate.

Other studies in the past have analyzed gene expression levels solely from peripheral blood indicating circulating tumor cells within this compartment. Most of them demonstrated significant impact on tumor recurrence or survival for patients positive for individual markers like *CK20* in CRC and other gastrointestinal cancers. However, in the majority these studies were based on a single marker level like *CK20* that detects differentiated circulating tumor cells [27,28].

*CD133* is one of the most frequently analyzed putative markers for CICs in patients with CRC, pancreatic cancer or hepatocellular carcinoma [29–35]. Clinical studies in patients with CRC have shown that, as in other solid cancers, high *CD133* expression profiles in peripheral blood were associated with decreased cumulative survival in patients in a metastasized tumor stage [36]. Another study investigated primary tumor tissue resections from a cohort of patients with G2-differentiated, pT2 and pT3 CRCs, without lymph node involvement. They demonstrated that high expression of *CD133* was an independent prognostic factor [33]. However, independent reports questioned the view of *CD133* being a universal marker for tumor initiating cells as *CD133*^−^ cells were also capable of tumorigenesis [37,38]. Interestingly, another functional marker that was not investigated in this study is the adhesion molecule CD44v6. It was described to confer the ability to colorectal stem cells to migrate at distant sites and develop CRC metastases [39]

Intestinal stem cells and a subpopulation of colon cancer cells were shown to express *Lgr5* [40–43]. It was also shown that *Lgr5* expression in selected UICC stage IV patients was associated with a poor prognosis [41]. Expression of *Lgr5* did not influence overall survival nor relapse probability in bone marrow although its expression significantly correlated with *CK20* expression. However, more recent evidence from a meta-analysis points to an association between *Lgr5* expression and prognosis [44]. This supports the herein described findings. Expression of the multidrug resistance mediator, *ABCB5*, in CICs has been characterized in previous studies on human melanoma [22,23] and hepatocellular carcinoma [45]. Recently, our group showed that *ABCB5* expression was significantly correlated with resistance to 5-FU chemotherapy in a xenotransplantation model of CRC [24]. *ABCB5* expression was also correlated with resistance to doxorubicin, camptothecin, and 5-FU in malignant melanoma and hepatocellular carcinoma. Moreover, we found overexpression of *ABCB5* in a subset of 5-FU-refractory cells in primary tumor specimens resected from patients with rectal cancer after neoadjuvant radio chemotherapy. Those results pointed to the clinical significance of *ABCB5* in CRC. We could show that *ABCB5* expression in the bone marrow from patients with CRC was strongly dependent on tumor grading and wall infiltration. It negatively influenced overall survival of the analyzed patients when detected in their bone marrow, indicating a cofactor for recurrent disease in these patients.

## Conclusion

Our data demonstrate a molecular signature expression for *ABCB5* and *Lgr5* as gene profile for cancer cells with specific characteristics for chemo resistance and self-renewal in the bone marrow of patients with CRC. These findings indicate that tumor cell migration can occur early during tumor progression, dependent on differentiation (grading) of the primary tumor (poorly versus well differentiated cancer cells), and that a subset of these migrating cells with specific self-renewal capacity and chemo resistance has significant negative influence on overall survival. This sheds light on prognostic significance of cancer initiating cell dissemination with future therapeutic implications in colorectal cancer.

## Figures and Tables

**Table 1:** Gene expression profiles in the bone marrow compartment from patients with colon cancer with respect to clinicopathological characteristics.

Variables	ABCB5		Lgr5		CD133		CK20	
	−	+	−	+	−	+	−	+
No.	4	41	2	44	11	35	17	29
**Age**								
<65	1	7	1	8	3	7	4	8
>65	3	34	1	36	8	28	13	21
Gender								
Male	2	17	0	17	5	12	7	12
Female	2	24	2	27	6	23	10	17
**Differentiation (Grading)**								
G1	2	0	1	1	0	1	2	0
G2	2	28	1	29	7	24	10	20
G3	0	13	0	14	4	10	4	9
**T category**								
T1	2	1	1	2	1	1	3	0
T2	1	2	0	3	1	2	3	0
T3	1	31	1	32	8	24	8	20
T4	0	8	0	8	1	8	3	9
**N category**								
N0	3	19	1	22	6	16	8	14
N1	0	11	1	10	3	7	4	7
N2	1	11	0	12	2	12	5	8
**M category**								
M0	3	31	2	33	10	25	14	21
M1	1	10	0	11	1	10	3	8
**UICC**								
I	2	5	1	6	3	5	6	2
II	1	15	1	16	6	11	9	5
III	1	9	0	10	1	9	1	9
IV	0	12	0	12	1	10	1	12

+ /−: Significant versus not Significant Upregulation of Each Gene of Interest; T: Tumor Size Categories; N: Nodal Involvement Categories; M: Distant Metastasis; UICC: Union for International Cancer Control.

**Table 2:** Univariate analysis of clinicopathological characteristics and investigated biomarkers in the bone marrow compartment characteristic of DTCs with respect to overall survival in the patients with colon cancer.

Variables	n (%)	Overall survival N (%)	P
Total	49 (100)	21 (42.9)	
**Age**			
< 65	11 (22)	9 (81.8)	0.026
≥ 65	38 (78)	7 (18.4)	
**UICC stage**			
I	8 (16)	7 (87.5)	0.025
II	17 (35)	8 (47.1)	
III	10 (20)	2 (20)	
IV	14 (29)	0 (0)	
**ABCB5**			
Positive	41 (82)	5 (12.2)	0.022
Negative	4 (8)	4 (100)	
**Lgr5**			
Positive	44 (90)	7 (15.9)	0.049
Negative	2 (4)	2 (100)	
**CD133**			
Positive	35 (71)	11 (31.4)	0.529
Negative	11 (22)	9 (81.8)	
**CK20**			
Positive	29 (59)	15 (42.9)	0.683
Negative	17 (36)	7 (41.2)	

UICC: Union for International Cancer Control.

**Table 3:** Clinicopathological characteristics of the analyzed patients with colon cancer (n=49).

Characteristics	No. of patients	%
Age, yr (Median: 68.7 yr; range 41–82 yr)		
< 65	11	22.4
≥ 65	38	77.5
**Gender**		
Male	19	38.8
Female	30	61.2
Tumor Location		
Colon	49	100
Rectum	0	0
**Tumor Differentiation**		
Well	2	4.1
Moderate	31	63.3
Poor	16	32.7
Mucinous	0	0
**UICC stage**		
I	8	16.3
II	17	34.7
III	10	20.4
…IV	14	28.6
**T category**		
T1	3	6.1
T2	3	6.1
T3	33	67.3
T4	10	20.4
**N category**		
N0	23	46.9
N1	11	22.4
N2	15	30.6
**M category**		
M0	35	71.4
M1	14	28.6
**Resection level of completeness**		
R0	34	69.4
R1	1	2
R2	14	28.6

UICC: Union for International Cancer Control; T: Tumor Size Categories; N: Nodal Involvement Categories; M: Distant Metastasis
